# High-altitude gastric disorders: insights into maladaptation pathophysiological mechanisms, disease spectrum and prevention strategies

**DOI:** 10.3389/fphys.2026.1944395

**Published:** 2026-10-12

**Authors:** Huihui Shang, Rong Zhang, Youbang Xie, Airong Yang, Jing Wang

**Affiliations:** 1 Department of Health Medicine, The 991st Hospital of the Joint Logistic Support Force of the People’s Liberation Army, Xiangyang, Hubei, China; 2 Department of Neurosurgery, The 991st Hospital of the Joint Logistic Support Force of the People’s Liberation Army, Xiangyang, Hubei, China; 3 Department of Hematology, Qinghai Provincial People’s Hospital, Xining, Qinghai, China; 4 Qinghai Provincial Hospital of Cardiovascular and Cerebrovascular Diseases (Qinghai Provincial Institute of High Altitude Heart Disease), Xining, Qinghai, China; 5 Department of Digestive Endoscopy, The General Hospital of Northern Theater Command, Shenyang, Liaoning, China

**Keywords:** chronic mountain sickness, gastric mucosal injury, high altitude, hypoxia-inducible factor, prevention, treatment

## Abstract

High-altitude environments are characterized by hypoxia (low oxygen levels), low atmospheric pressure, and intense ultraviolet radiation. Researchers have identified these factors as key contributors to gastric damage. Environmental stressors compromise the gastric mucosal barrier by causing direct damage, oxidative stress, inflammatory responses, and tight junction disruption. Furthermore, gastrointestinal hormones and motility dysfunction, neuroendocrine disturbances, and gut microbiota dysbiosis increase intestinal permeability and immune imbalance. These factors can trigger gastric diseases and multi-organ dysfunction. This article highlights the role of bidirectional regulation of the HIF signaling pathway and its metabolic reprogramming cascades in high-altitude gastric diseases. While previous reviews have primarily focused on intestinal damage, this review centers on the stomach, elucidating the pathophysiological mechanisms of gastric damage caused by failed hypoxia acclimatization to high altitudes. It covers the progression from acute damage to chronic lesions (e.g., gastric damage associated with high-altitude polycythemia (HAPC)) and the pathophysiological mechanisms of malignant transformation. It also discusses associated diseases such as acute gastric mucosal injury, peptic ulcers (which carry an increased risk of bleeding and perforation), and an elevated prevalence of gastroesophageal reflux disease (GERD). Long-term exposure may also increase the risk of gastric cancer. Prevention and standardized treatment of high-altitude gastric disorders are of critical importance.

## 1 Introduction

Approximately 500 million people live above 1,500 m, and millions more travel there annually (Tremblay and Ainslie, 2021). Long-term residents and migrants in these regions face significant health challenges due to various factors, such as low atmospheric pressure, oxygen deficiency, cold temperatures, and intense ultraviolet radiation. This elevated the risk of chronic mountain sickness (CMS) (West, 2016; Cui et al., 2022; Wang et al., 2022). In acute altitude situations, individuals unaccustomed to high-altitude environments are susceptible to acute mountain sickness (AMS) when they are suddenly exposed to low pressure and oxygen-deprived conditions (Bärtsch and Swenson, 2013). The gastric mucosa is particularly vulnerable due to its high metabolic demand and sensitivity to ischemia. Gastrointestinal symptoms (anorexia, nausea, abdominal pain) are among the earliest and most common complaints at altitude (Luks et al., 2017; Forrer et al., 2023). Epidemiological data indicate a marked increase in the incidence of gastrointestinal disorders at elevations above 2,500 m (Fruehauf et al., 2020).

Previous reviews have reported that a stay at high altitude may lead to gastrointestinal discomfort due to acute hypoxia. Hypoxic stress is a key factor in human behavior at high altitudes. Hypoxia can lead to bacterial translocation and gastrointestinal barrier dysfunction, thereby triggering “leaky gut syndrome”. The major causes of gastrointestinal discomfort are increased intestinal permeability, inflammation, and microbial dysbiosis. In this condition, the tight junctions that guard the anti-lumen space of the body become leaky or loose, allowing an uncontrolled exchange of harmful molecules into the bloodstream and causing local or systemic inflammation (Tulsawani and Vohora, 2023). Therefore, acute exposure to hypobaric hypoxia may result in gastrointestinal injury and damage to the mucosal epithelium of the intestine. However, researchers have discussed the specific pathophysiology of the gastric mucosa relatively little. This review systematically examines the maladaptive pathophysiological mechanisms and a spectrum of gastric diseases. It also proposes corresponding preventive and therapeutic measures, aiming to develop new treatment strategies for patients with high-altitude gastric disorders.

## 2 Pathophysiological mechanisms

The combination of low atmospheric pressure and low oxygen levels at high altitudes can cause varying degrees of damage to the gastric mucosa. The mechanisms underlying gastric maladaptation at high altitudes are extremely complex and involve acute and chronic forms. The primary mechanism involves hypoxia-induced responses. In addition to the classic pathways, including oxidative stress, inflammatory responses, neuroendocrine dysfunction, and gut microbiota dysbiosis, this mechanism encompasses cutting-edge HIF signaling pathways, multi-omics differential biomarkers, and emerging molecular evidence. However, some of these mechanisms lack biomarkers that are specific to changes in the stomach in high-altitude environments, as well as emerging molecular evidence. We will discuss these topics in turn below.

### 2.1 Core maladaptation pathophysiological mechanisms

#### 2.1.1 Gastric mucosal barrier disruption

The mucosal barrier integrity is crucial for homeostasis, nutrient absorption, flora balance, and toxin blockade (Cheng et al., 2024). Acute high-altitude exposure impairs barrier function, and prolonged hypoxia exacerbates damage via immune, microbiota, and mucosal layer alterations. Physical exertion worsens injury by reducing splanchnic circulation (McKenna et al., 2022). Mechanisms include direct hypoxic injury, oxidative stress, inflammation, and tight junction disruption, each discussed below with specific molecular details.

##### 2.1.1.1 Direct hypoxic injury

Prolonged hypoxia leads to gastric mucosal ischemia. Gastric blood flow velocity decreases, and the partial pressure of oxygen within the gastric wall drops. This reduces epithelial cell regeneration and increases bile reflux. The severity of injury correlates with exposure duration. Hypoxia-induced damage primarily affects the stomach and small intestine (Wang et al., 2024). Specifically, peripheral vasoconstriction occurs under hypoxia, diverting blood away from splanchnic beds and further compromising mucosal oxygenation. Animal studies have confirmed that hypoxia alone can cause damage to the gastric mucosa in rats, with lesions almost exclusively located in the gastric body and classified as type UL-1 erosion (Nakazawa et al., 1980). Hypoxia can directly reduce levels of the protective prostaglandins PGE2 and PGI2 in the gastric mucosa, thereby weakening the mucosal defense barrier (Goto et al., 1987). Furthermore, hypoxia disrupts the gastric microecological balance, leading to inflammatory responses, alterations in the mucus layer, and destabilization of the gastric microbiota (Chen et al., 2025), thereby exacerbating local damage. Collectively, these findings indicate that hypoxia causes direct and persistent damage to the gastric mucosa via a multilevel pathway involving reduced blood flow, insufficient oxygen supply, depletion of barrier molecules, and disruption of the microecological balance.

##### 2.1.1.2 Oxidative stress

The gastrointestinal tract is a significant source of reactive oxygen species (ROS). Excessive ROS production can disrupt the body’s homeostasis and lead to oxidative tissue damage. ROS include radical compounds such as superoxide (O_2_^−^) and hydroxyl radicals (HO·), as well as non-radical derivatives including hydrogen peroxide (H_2_O_2_), singlet oxygen (1O_2_), and lipid hydroperoxides. In addition, myeloperoxidase-catalyzed reactions produce hypochlorous acid (HOCl) and chloramines (RNHCl), which represent reactive chlorine species that may also be generated under hypoxic/inflammatory conditions. Reactive nitrogen species (RNS) include nitric oxide (·NO), nitrogen dioxide (·NO_2_), and non-radical compounds such as peroxynitrite (ONOO^−^) and dinitrogen trioxide (N_2_O_3_) (Bhattacharyya et al., 2014). High-altitude-linked hypobaric hypoxia is considered the main cause of ROS production. If ROS production increases and the body’s natural antioxidant system (including superoxide dismutase (SOD), catalase, and glutathione peroxidase) cannot counteract this, the ROS can damage biomolecules in cells, including lipids, proteins, and DNA. This can sometimes result in cell death, leading to mucosal cell injury and barrier disruption. This condition is known as oxidative stress. RNS are often associated with ROS, and under low-pressure hypoxic conditions, RNS formation can exceed the body’s defenses, leading to oxidative stress. Oxidative stress contributes to various gastric diseases, including gastroduodenal ulcers and gastric cancer (Li et al., 2024). ROS upregulate hypoxia-inducible factor-1α (HIF-1α), resulting in the expression of several genes that regulate tumor growth and fuel ongoing tumor progression (Hielscher and Gerecht, 2015). At the same time, HIF-1α alters metabolism toward glycolysis, modulating ROS and initiating inflammatory pathways. HIF-1α activates Drp1, leading to mitochondrial fission. This increases ROS levels and activates the NOD-like receptor protein 3 (NLRP3) inflammasome, worsening mucosal damage (Xiao et al., 2024). Enhanced glycolysis provides ATP and biosynthetic precursors, but it can also fuel ROS production through NADPH oxidase activation and mitochondrial electron leakage. This establishes a feed-forward loop that amplifies oxidative damage under sustained hypoxic stress (Liu et al., 2020; Ucaryilmaz Metin and Ozcan, 2022). Human multi-omics data from mountaineers confirm that extreme-altitude exposure upregulates glycolysis and antioxidant gene expression (orchestrated by HIF1α and NFE2L2), alongside enhanced glutamine and fatty acid metabolism (Yin et al., 2025). These metabolic adaptations, while initially protective, may become maladaptive under sustained hypoxic stress, perpetuating oxidative injury to the gastric mucosa.

Consequently, the following cascade occurs: hypoxia → ROS↑→ HIF↑ → glycolytic metabolic reprogramming → ROS↑.

##### 2.1.1.3 Inflammatory response

Oxidative stress triggers the release of tumor necrosis factor-α (TNF-α), platelet-activating factor (PAF), interleukin-1 (IL-1), and interleukin-6 (IL-6). These increase vascular permeability, recruit neutrophils, and further reduce microcirculation (Gao and Huang, 2016; El Alam et al., 2022). TNF-α plays a crucial role in gastric mucosal injury and also promotes the secretion of IL-1, IL-2, and IL-6. IL-1 is a key inflammatory cytokine involved in regulating immune responses and inflammatory processes. PAF is one of the more potent inflammatory mediators that cause gastric mucosal injury. It increases vascular permeability, induces vasoconstriction and hypotension, reduces gastric mucosal blood flow, and synergizes with other inflammatory cytokines. While direct human gastric data are limited, animal studies have found that exposure to acute hypobaric hypoxia (HH) increase zonulin, TNF-α, IL-1β and IL-6 serum levels, increases the CD4^+^/CD8^+^ T cell ratio, increases the small intestine NK cell population, and increases the expression levels of small intestine TLR4, NF-κB, HIF-1α, and inducible nitric oxide synthase (iNOS) mRNA and protein. Conversely, it decreases occludin mRNA and protein expression levels (Wang and Huo, 2021). Researchers established an inflammatory model using lipopolysaccharide and conducted animal experiments to investigate the impact of hypoxia on the colonic TLR4/NF-κB signaling pathway and its associated inflammatory cytokines, including TNF-α, IL-1β, IL-6 and interferon-γ (IFN-γ). The results suggest that hypoxia could result in substantial damage to the colonic mucosa and impair the TLR4-mediated innate immune response in the colon, potentially reducing host defense responses to colonic pathogens (Han et al., 2023). Hypoxia is mediated by the specific overexpression of HIF-1α in the distal colon, which induces markedly different changes in the proximal and distal colon. The core mechanism involves a synergistic interaction between hypoxia and Wnt signaling, forming a “self-reinforcing redox loop” in the distal colon that promotes stem cell proliferation. In contrast, the absence of this mechanism in the proximal colon results in a colon segment-specific hypoxic response (Chen et al., 2025). This is due to systemic inflammatory patterns (e.g., elevated TLR4/NF-κB), which are likely to be operative in the stomach given the shared vascular and immune pathways. TNF-α, IL-1β, and IL-6 levels were all significantly elevated in the gastric tissue of mice with hypoxia and Helicobacter pylori (*H. pylori*) infection, exacerbating the infection-induced inflammatory response. This may be due to the inhibition of the Nrf2 signaling pathway in gastric tissue (Li et al., 2026).

##### 2.1.1.4 Tight junction disruption

Maintaining the integrity of the gastric mucosal barrier is crucial for gastric homeostasis, and the epithelial barrier - composed of epithelial cells interconnected by tight junctions - is its most important component. Tight junctions include: zonula occludens (ZO)-1 and its associated cytoplasmic scaffolding proteins ZO-2 and ZO-3; cingulin; MARVEL proteins associated with tight junctions (occludin, tricellulin, and MARVELD3); and tight junction proteins. ZO-1 and occludin, in particular, participate in cytoskeletal formation and are important structural components of tight junctions (Zuo et al., 2020). Animal studies have shown that, in mice exposed simultaneously to high-altitude hypoxia and *H. pylori* infection, the expression levels of ZO-1, occludin, and Bcl-2 in gastric tissue decrease, while the expression levels of Bax and caspase-3 increase. These findings confirm that high-altitude hypoxia exacerbates *H. pylori* infection, further compromising the gastric mucosal barrier by reducing tight junction protein expression and inducing changes in apoptosis-related protein expression, thereby leading to more severe gastric mucosal damage (Li et al., 2026). Furthermore, animal experiments demonstrated that after 3 hours of hypoxia exposure, the intestinal villi structure in the jejunum becomes impaired, Claudin-2 levels decrease, and crypt depth changes. In the colon, Claudin-1 levels decrease, and intercellular resistance is correspondingly reduced (Fedorova et al., 2025). Disruption of the tight junctions between the jejunum and the colon may lead to an imbalance in the gut microbiota, which can trigger a range of symptoms. We discuss these in detail in the chapter on “2.4 Gut microbiota dysbiosis”.

Claudin-18.2 is an isoform of a tight junction protein that is responsible for maintaining gastric homeostasis and protecting epithelial cells from low pH conditions. Its expression is strictly restricted to the stomach, and it regulates itself by activating the aPKC/MAPK/AP-1 pathway, making Claudin-18.2 an ideal tumor marker. It has also emerged as a promising therapeutic target in gastric cancer (Jin et al., 2024; Nakayama et al., 2024; Poniewierska-Baran et al., 2025). Claudin-18.2 maintains tight junction integrity in gastric cancer cells through interaction with ZO-1. Its downregulation disrupts cell-cell junctions, weakens epithelial barrier function, and promotes cell migration and invasion, whereas compensatory ZO-1 overexpression can partially reverse these effects (Li et al., 2025).

#### 2.1.2 HIF signaling pathway- a central regulatory hub

Building on the previously discussed mechanisms of direct hypoxic injury, oxidative stress, inflammatory responses, and tight junction disruption, this section focuses on the HIF signaling pathway. As a central regulatory hub, this pathway integrates these various injury mechanisms and plays a bidirectional regulatory role in how the gastric mucosa adapts to and responds to hypoxic injury.

The HIF gene family comprises HIF-1, HIF-2, and HIF-3. HIF plays a vital role in maintaining mucosal homeostasis by regulating various metabolic, inflammatory, and barrier-related pathways (Kim et al., 2021). All three HIFs consist of a unique, oxygen-sensitive α-subunit and a stable, common β-subunit. The most important member of the HIF family has two subunits: HIF-1α and HIF-1β. Under normoxic conditions, prolyl hydroxylases (PHDs) degrade HIF-1α. Conversely, in hypoxic environments, PHD activity is suppressed, leading to the stabilization of HIF-1α and the subsequent activation of multiple downstream target genes. Stabilized HIF-1α has been shown to enhance barrier integrity and mitigate inflammation by upregulating targets such as trefoil factors, CD73, E-cadherin, ATG9A, and various mucins (Dowdell et al., 2020; Hoffmann, 2021; Knutson et al., 2021). However, other evidence suggests that under high-altitude hypoxic conditions, excessive HIF-1α activation can induce iNOS expression and promote nitric oxide (NO) production. While this may improve microcirculation in the short term, it can lead to a redox imbalance over time (Zhang et al., 2015b; Wang et al., 2020). Furthermore, HIF-1α binds directly to the TLR4 promoter, activates the TLR4/MyD88/NF-κB signaling pathway, induces the release of proinflammatory cytokines, and exacerbates mucosal damage (Kim et al., 2010; Xu et al., 2014). A positive feedback loop between HIF-1α and NF-κB perpetuates inflammation (Zhang et al., 2012). HIF-1α expression is also regulated by the PI3K/AKT/NF-κB axis (Wan et al., 2014). Chronic HIF-1α also reduces goblet cell numbers, thereby weakening the mucus layer (Zhang et al., 2025). HIF-1α promotes vascular endothelial growth factor (VEGF) and metabolic reprogramming, aiding angiogenesis and immune evasion (Paredes et al., 2021). HIF-1β (also known as ARNT), the constitutive partner of the HIF-1 heterodimer, is essential for the transcriptional activity of HIF-1α. Loss of HIF-1β disrupts this complex and impairs its downstream effects. In particular, HIF-1β deficiency has been shown to impair claudin-1 localization and compromise tight junction architecture (Saeedi et al., 2015). HIF-1β-deficient mice exhibit more severe hypoxemia, greater weight loss, and an increased cardiac workload when exposed to hypoxia (Han et al., 2020).

HIF-2 is composed of HIF-2α and HIF-2β. HIF-2α is the main functional subunit, and it is abundant in tissues such as vascular endothelial cells and fetal lung fibroblasts. After HIF2-α is activated, it binds to ARNT to form a heterodimer. This heterodimer then specifically binds to the hypoxia response element of hypoxia-inducible factor (5’-TACGTGCG-3’), thereby upregulating the expression of these genes (Silagi et al., 2021). Researchers currently know that HIF-3, a less-studied member of the HIF gene family, is composed of HIF-3α and HIF-3β. The HIF-3α gene produces a variety of HIF-3α variants, and it is expressed and differentially regulated by hypoxia and other factors. Full-length HIF-3α protein functions as an oxygen-regulated transcriptional activator (Duan, 2016).

The expression kinetics of HIF isoforms differ: HIF-1α peaks within 4 h of hypoxia and subsequently declines, whereas HIF-2α peaks around 8 h and remains elevated for at least 24 h (Jaśkiewicz et al., 2022). These findings suggest that HIF-2 may play a more prominent role in long-term adaptation to chronic hypoxia. However, the regulatory mechanisms governing the transition between HIF-1 and HIF-2 dominance remain unclear. HIF-1α peaks early (acute response); HIF-2α dominates chronic adaptation. HIF-1α peaks early (acute response); HIF-2α dominates chronic adaptation. This “acute bidirectional → chronic metabolic remodeling” framework explains the progression from reversible injury to organic lesions and potentially to malignancy.

#### 2.1.3 Gastrointestinal hormones and motility dysfunction

The effects of acute high-altitude exposure on gastrointestinal hormones are complex and inconsistent. Gastrin and motilin levels increase, with a direct correlation between increased motilin levels and gastrointestinal symptoms. But the release of cholecystokinin, pancreatic polypeptide, and vasopressin is suppressed (Riepl et al., 2012). Endothelin-1 (ET-1) is a potent vasoconstrictor peptide. ET-1 levels increased in the blood under systemic hypoxia could lead to decreased mucosal blood flow and mucosal ischemia. Mucosal ischemia could activate pepsinogen and breach the gastric mucosal barrier, inducing mucosal lesions and subsequent gastric ulcers (Yang et al., 2022). This causes plasma to infiltrate the gastric lumen, thereby increasing blood viscosity. Concurrently, hypoxia induces erythrocytosis, which exacerbates blood viscosity further and promotes thrombus formation and hemorrhage. Consequently, gastric mucosal cells undergo ischemia-induced erosion, ulceration, and bleeding. Hypoxia specifically and potently inhibits gastric contractility by activating inhibitory pathways of the vagal nerves, resulting in severe motility impairment and delayed gastric emptying. Retained gastric contents cause distension, which triggers satiety through neural feedback and suppresses appetite. In severe cases, this can induce nausea (Yoshimoto et al., 2004).

Interstitial cells of Cajal (ICCs), which are found in the gastrointestinal tract, play a major role in gastrointestinal motility (Al-Sajee and Huizinga, 2012). Loss or dysfunction of ICCs causes gastrointestinal motility disorders. Oxidative stress damages ICCs, disrupting slow waves (Joung et al., 2021). *In vitro* experiments have identified TNF-α, IL-6, and NO as factors that impair ICCs. In inflammatory gastrointestinal diseases, the number of ICCs decreases, and they exhibit abnormal ultrastructure and impaired pacemaker function. This strongly suggests that gastrointestinal dysmotility in these diseases is partly due to ICC injury (Kaji and Hori, 2023). Therefore, oxidative stress and inflammatory responses caused by high-altitude hypoxia can both damage ICCs and affect gastrointestinal function.

#### 2.1.4 Gut microbiota dysbiosis

The gastrointestinal tract is home to a complex and dynamic community of microorganisms. These microbial communities regulate homeostasis and host immune responses, thereby influencing health, energy metabolism, and epithelial integrity, and promoting overall well-being (Kayama and Takeda, 2020; Sun et al., 2020; Debnath et al., 2021; Smet et al., 2022; Wang et al., 2023b). The species composition of the intestinal microbiota is sensitive to environmental temperature; a decrease in environmental temperature leads to reduced α-diversity of the microbiota (Chevalier et al., 2015; Moreno-Navarrete and Fernandez-Real, 2019; Su et al., 2024). At high altitude, faecal microbiota analysis revealed a significant decrease in total aerobes and an increase in total and facultative anaerobes (Kleessen et al., 2005; Adak et al., 2013). This disruption to the balance between beneficial and pathogenic gut bacteria can indirectly induce or exacerbate gastrointestinal diseases (Adak et al., 2014; Karl et al., 2018). Notably, butyrate-producing bacteria (e.g., Lachnospiraceae and Clostridiaceae) decline at high altitudes, thereby reducing short-chain fatty acids (SCFAs) (Wang et al., 2022; Zhao et al., 2024). Butyrate is the favored energy source for colonic epithelial cells and plays a vital role in maintaining intestinal barrier integrity and regulating immune responses (Dengler et al., 2021). Butyrate inhibits NF-κB/NLRP3 and downregulates HIF-1α, enhances tight junction protein occludin expression in Caco-2 cells, and restores the decreased permeability of the Caco-2 monolayer due to lipopolysaccharide (Bakshi and Mishra, 2025). Mechanistic validation experiments revealed that butyrate, a metabolite produced by Firmicutes bacteria, significantly downregulates the protein expression of HIF-1α, GCK, PFK, PKM, and LDH, thus inhibiting the HIF-1α/glycolytic pathway (Yan et al., 2024). Therefore, gut microbiota dysbiosis caused by high-altitude hypoxia leads to tight junction disruption and activation of the HIF-1α/glycolysis pathway, resulting in gastric mucosal damage.

In high-altitude environments with low atmospheric pressure and oxygen levels, a series of reactions within the body can damage the gastric mucosa. This process involves the following steps: hypoxia → HIF activation → glycolytic metabolic reprogramming → mucosal energy deficiency → tight junction disruption.

#### 2.1.5 Neuroendocrine dysregulation

Rats exhibited significant alterations in gut microbiota, the hypothalamic-pituitary-adrenal (HPA) axis, and hypothalamic-pituitary-thyroid (HPT) hormones at simulated 5,500 m altitude, particularly during the acute phase (Xie et al., 2020). Acute exposure to high altitude significantly activates the HPA axis in male rats while suppressing thyrotropin-releasing hormone (TRH) and tT44 in the HPT axis (Hao et al., 2015; Wang et al., 2023a). Neuroendocrine dysfunction associated with acute hypoxic stress may trigger reduced appetite (Gotoh et al., 2013). Under high-altitude hypoxic stress, hypothalamic dysfunction and excessive sympathetic nervous system excitation can lead to the secretion of corticosteroids, which promote gastric acid and pepsin secretion. This disrupts the secretion of gastrointestinal motility hormones, causing gastrointestinal motility dysfunction and damaging the digestive tract mucosal barrier, ultimately resulting in erosions and ulcer formation (Mönnikes et al., 1993; Zhang et al., 2015a).

### 2.2 Chronic maladaptive mechanisms

For individuals who live at high altitudes for extended periods or repeatedly travel to such environments, the pathological processes described above will persist and interact. This could potentially lead to chronic maladaptive responses that are more characteristic of the environment and have long-term consequences.

#### 2.2.1 High-altitude polycythemia and gastric injury

In high-altitude regions, prolonged tissue hypoxia stimulates the production of erythropoietin (EPO), leading to excessive erythrocyte proliferation and a significantly elevated hemoglobin concentration-a condition clinically defined as HAPC (Kasperska and Zembron-Lacny, 2020; Yang et al., 2021). The pathogenesis of HAPC is complex and involves multiple interconnected mechanisms. In the stomach, ROS upregulates HIF-1α expression, and elevated HIF-1α levels are closely associated with HAPC-related gastric mucosal lesions in Tibetans living at high altitudes (Li and He, 2019). Hypoxia stabilizes HIF, which in turn drives EPO production and triggers a cascade of inflammatory responses, reduced antioxidant capacity, and increased free radical generation, ultimately culminating in HAPC (Tang et al., 2024). Additionally, genetic polymorphisms contribute to individual susceptibility to HAPC (Zhang et al., 2022).

Prolonged hypoxic exposure reduces oxygen delivery to systemic tissues and organs, with microcirculatory impairment being particularly pronounced, predisposing HAPC patients to multi-organ and multi-system damage. The gastrointestinal tract, being highly sensitive to hypoxia, undergoes pronounced physiological and structural alterations. In severe cases, this can lead to gastric mucosal erosion, ulceration, necrosis, or even malignant transformation (Nayak et al., 2020). Patients with HAPC exhibit higher erythrocytosis, blood viscosity, and platelet activation, which may increase the incidence of thrombosis (Wang et al., 2023). At the cellular level, HAPC induces excessive proliferation of gastric parietal cells, resulting in hyperacidity, while simultaneously promoting significant apoptosis of gastric mucosal cells and reducing overall cell numbers. Moreover, erythrocytes are distributed not only within the vascular compartment but also in the interstitial spaces of gastric tissue, further increasing the risk of bleeding (Li et al., 2017).

At the systemic level, hypoxia at high altitudes reduces IL-10 and IL-22 levels, which in turn decreases hepcidin levels, leading to an excess of available iron that promotes erythropoiesis and contributes to HAPC pathogenesis in susceptible individuals (Liu et al., 2018). Plasma levels of IL-6, IL-8, and TNF-α are elevated in HAPC patients, accompanied by oxidative stress and impaired NO production (Brewster et al., 2021). Proteomic analyses have further revealed that differentially expressed proteins in HAPC patients are primarily enriched in inflammatory and immune response pathways (Wang et al., 2019). In response to HAPC-induced gastric mucosal lesions, the gastric mucosa initiates protective responses by upregulating APOA4 and APOC3 to suppress gastric acid secretion and inflammation. Conversely, it downregulates the gastric inhibitory factor (GIF) gene, which induces anemia and counteracts erythrocytosis (Li et al., 2015). This adaptive response may lead to adverse effects, including metabolic disorders, anemia, and malnutrition.

#### 2.2.2 Synergistic stressors (cold, diet, UV, psychology)

High-altitude environments often cause damage to the gastrointestinal tract due to the combined effects of hypoxia and other environmental and lifestyle factors. Physiological stressors such as hypoxia and cold can activate the sympathetic-adrenal medullary system, reducing blood supply to the gastric mucosa and inducing acute gastric mucosal lesions in rats (Ye et al., 2013). Residents of Tibetan regions typically consume a monotonous diet featuring minimally processed foods high in salt and fat, such as raw beef and mutton, and do not consume enough fruit and vegetables. Experimental studies confirm that high-salt diets induce chronic atrophic gastritis and intestinal metaplasia in rats (Yin et al., 2021). Compared to lowland populations, these dietary habits slow gastrointestinal mucosal blood flow, weaken the regenerative capacity of epithelial cells, and reduce mucus secretion. This makes high-altitude populations more susceptible to gastric mucosal damage (Negi et al., 2013). In addition to cold and dietary factors, UV radiation, though primarily affecting the skin, can induce systemic oxidative stress and immunosuppression, which may indirectly impair gastric mucosal defense. Psychological stress, which is common among plateau migrants, activates the HPA axis and the sympathetic nervous system. These further compromise mucosal blood flow and repair mechanisms. Psychological stressors regulate physiological processes involving the nervous, endocrine, and immune systems, and are closely associated with the onset and progression of cardiovascular and gastric diseases (Oroian et al., 2021).

**Figure 1** Illustrates the pathogenesis of high-altitude-related gastric disorders.

**Figure 1:**
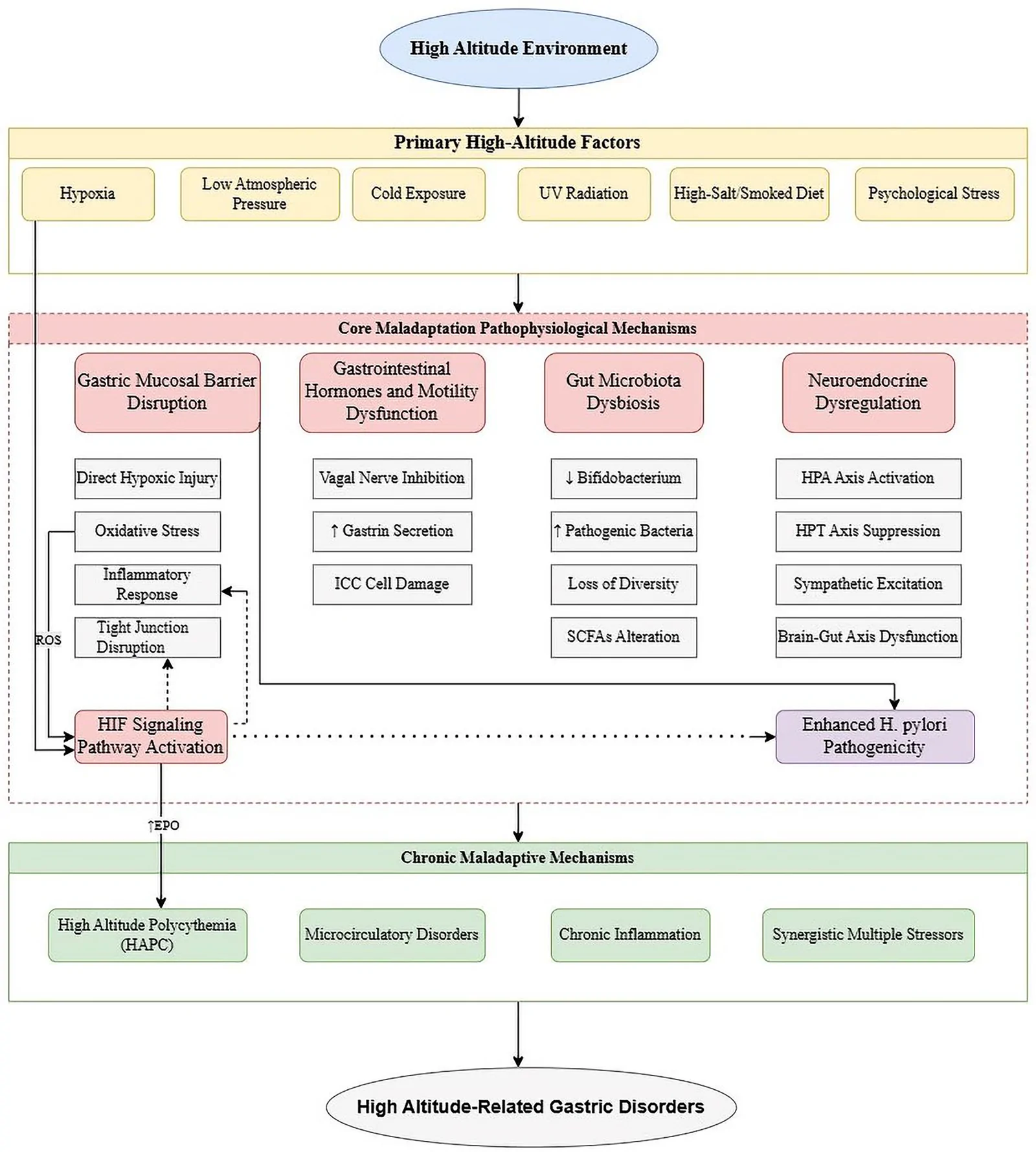
Pathogenesis of altitude-related gastric disorders.

## 3 Clinical spectrum and evidence evaluation

Ultimately, the superimposition of acute and chronic pathological mechanisms manifests clinically as a series of specific altitude-related gastric disorders. In the following subsections, we explicitly link each disorder to the pathophysiological mechanisms.

### 3.1 Acute mucosal injury and peptic ulcer

Acute gastric mucosal injury is a common digestive system disorder. Under normal physiological conditions, protective factors in the gastric mucosa (such as gastrin, prostaglandins, and neurokinin A) protect the mucosa from erosion and damage caused by aggressive agents such as gastric acid, pepsin, and bile salts. However, when the body enters an abnormal state, these aggressive factors intensify, and protective factors diminish. This reduces the mucosa’s resistance and impairs the barrier function of bicarbonate and mucus, thereby causing acute gastric mucosal injury. Hypoxia can directly damage the gastric mucosa by causing oxidative stress, disrupting tight junctions (which leads to increased permeability), and impairing microcirculation. It can also promote hyperacidity and motility disorders. These combined effects result in acute erosions and ulcerations. High-altitude stress ulcer (HSU) is a major clinical challenge. Among 13,502 railway workers at an altitude of 4,905 m, the incidence of gastrointestinal bleeding was 0.49%, increasing with altitude (Wu et al., 2007). Endoscopic studies of 25 mountaineers showed an increase in lesion incidence from 12% at baseline to 60.9% on day 4 and an increase in ulcer incidence from 0% to 21.7% (Fruehauf et al., 2020). HAPC increases the risk of bleeding 5.27-fold (Xu and Ci-ren-yang-jin, 2022). Between September 2017 and August 2018, a field hospital reported 5 cases of gastric antral perforation among soldiers deployed to altitudes exceeding 15,000 ft, which was attributed to atrophic gastric mucosa with antral gastritis. This condition can potentially cause increased intraluminal pressure and local ischemia under hypoxic conditions (Pawar et al., 2018). Another study documented cases of Mount Everest trekkers halting their journey due to acute abdominal pain (perforated peritonitis), who were ultimately diagnosed with gastric perforations. These cases suggest that reduced atmospheric pressure at high altitudes may cause excessive distension of hollow viscera, potentially triggering perforation (Amatya et al., 2018).

### 3.2 *H. pylori* infection

The global prevalence of *H. pylori* infection is 50% (Hooi et al., 2017). Chronic *H. pylori* infection can lead to diseases such as gastritis, peptic ulcers, gastric mucosal atrophy, and gastrointestinal mucosal metaplasia. These can ultimately result in gastric cancer or mucosa-associated lymphoid tissue (MALT) (Sugano et al., 2015). Due to variations in the bacterium’s pathogenicity, mechanisms of action, and interaction with the host immune system and environmental factors, the consequences of *H. pylori* infection can differ from person to person (Malfertheiner et al., 2023). Compared with gastric epithelial cells (GECs) under normal oxygen concentrations, GECs in hypoxic environments exhibit significantly increased susceptibility to *H. pylori* infection (Poirah et al., 2025). *H. pylori* infection is more prevalent among residents of high-altitude regions than low-altitude regions, with patients in high-altitude regions exhibiting more severe cases of *H. pylori*-associated gastritis (Sharma et al., 2022). A multicenter study in Peru found that *H. pylori* infection prevalence was higher among residents of high-altitude areas (86.9%), and that patients in these areas exhibited more severe pathological findings, with higher proportions of chronic deep gastritis and atrophic gastritis (Requena et al., 2024). In high-altitude regions, patients with *H. pylori*-associated gastritis exhibit a higher prevalence of diffuse erythema and widened folds. Pathological analysis reveals elevated gastric mucosal inflammation scores and more severe chronic and active inflammation in these patients. Furthermore, patients with *H. pylori*-associated gastritis in high-altitude regions exhibit elevated serum levels of TNF-α, IL-1β, IL-6, and MDA, alongside reduced SOD and GSH-Px activity. The hypoxic environment at high altitudes exacerbates the oxidative stress and inflammatory response caused by *H. pylori* infection. It also reduces tight junction protein expression and triggers changes in protein expression associated with apoptosis. These further compromise the gastric mucosal barrier, leading to more severe gastric mucosal damage (Li et al., 2024).

Therefore, enhanced *H. pylori* screening and treatment among high-altitude residents is necessary to reduce the prevalence and severity of chronic gastritis. Additionally, antioxidant therapy may be an effective way to treat *H. pylori*-associated gastritis in high-altitude regions.

### 3.3 Gastroesophageal reflux disease

The gastrointestinal tract is a common target organ for high-altitude sickness and frequently presents symptoms such as vomiting and abdominal pain. These symptoms fall within the spectrum of gastroesophageal reflux (GER) (Guo et al., 2022; Mishra and Bakshi, 2023). Research has confirmed that GER symptoms triggered by physiological or pathological factors increase the risk of developing GERD, while nutritional interventions can reduce its incidence (Bischoff et al., 2022). AMS is often accompanied by headaches, and the increased frequency of vomiting induced by headaches may lead to damage to the cardia and oesophageal mucosae, which are key factors in inducing GERD (He et al., 2022). Further research indicates a significant correlation between altitude and GERD risk, with a prevalence rate of 10.8% in Tibet, China (Zhang et al., 2019). GERD incidence in Golmud, Qinghai (9.41%) is higher than in lowland areas such as Beijing (5.77%), Shanghai (5.77%), and Guangdong (2.3%) (Yao et al., 2018). A population-based study conducted in high-altitude regions of India revealed a relatively high GERD prevalence of 18.7% (Kumar et al., 2011). Mechanistically, hypoxia-induced delayed gastric emptying increases intragastric pressure and promotes reflux, while mucosal barrier disruption renders the esophagus more susceptible to acid injury.

### 3.4 Gastric cancer

#### 3.4.1 Epidemiological evidence

Gastric cancer is a common malignant tumor worldwide, with millions of cases diagnosed annually. It exhibits alarming incidence and mortality rates (Bray et al., 2024). There is a growing body of evidence suggesting that exposure to high altitudes is a significant risk factor for gastric cancer (Mamun et al., 2024). The strongest evidence to date comes from a meta-analysis (random-effects model) which included 5,963 cases. This analysis revealed a notably higher risk of gastric cancer among individuals residing in high-altitude regions, with a pooled hazard ratio (HR) of 1.89 (95% CI: 1.43-2.52) (Montalvan-Sanchez et al., 2022). Some studies have reported variations in gastric cancer incidence rates by altitude, with higher rates observed at higher altitudes (Bonequi et al., 2013). Evidence suggests a positive association between altitude and gastric cancer risk in Latin America (Torres et al., 2013), and in Ardabil, Iran, gastric cancer incidence rises with increasing altitude (Amani et al., 2015). Living at high altitude has been associated with a high mortality rate from gastric cancer. The odds ratio (OR) for dying from gastric cancer was also found to be significantly higher (OR: 1.204; p<0.001) (Garrido and Garrido, 2018). High altitude has been confirmed as an independent prognostic risk factor for gastric cancer. Multivariate Cox regression analysis showed that residents living at high altitudes were significantly more likely to die from gastric cancer (HR = 1.50; 95% CI: 1.06-1.82; p = 0.018), an effect independent of other clinical and pathological variables (Yin et al., 2020).

#### 3.4.2 Pathophysiological mechanisms

The unique environment of hypoxia and low atmospheric pressure may influence tumor microenvironment (TME) remodeling and metabolic reprogramming by inducing chronic hypoxic adaptation in the body. These factors contribute to regional variations in gastric cancer biology and treatment response (Castillo et al., 2019). Characteristic features of TME include hypoxia, metabolic dysregulation, and immunosuppression. Adaptation to hypoxia, metabolic dysregulation, and phenotypic shift in immune cells via N6-methyladenosine (m^6^A) methylation synergistically promotes the formation of an immunosuppressive TME that supports tumor proliferation and metastasis (Zhang et al., 2022). At the molecular level, chronic hypoxia related to altitude can induce various genetic and epigenetic changes. These include abnormalities in microRNAs, the p53 protein, and lymphocyte activity. This is accompanied by the downregulation of pro-apoptotic molecules such as Fas ligand and the upregulation of pro-metastatic factors such as VEGF and HIF (Calderón-Gerstein and Torres-Samaniego, 2021).

Regarding upstream driving mechanisms, single-cell RNA sequencing (scRNA-seq) has confirmed that tumor cells and stromal cells in gastric adenocarcinoma exhibit significant hypoxic characteristics driven by the transcription factors EHF, EIF1AD, GLA, KEAPI, and MAGED2 (Yang et al., 2025). EHF overexpression, for example, activates HER2 signaling and the downstream MAPK/Erk and PI3K/AKT pathways (Shi et al., 2016), and pathways such as HER2/PI3K-AKT can upregulate HIF-1α (Keld and Ang, 2011), suggesting the existence of an “EHF → HER2 → PI3K-AKT/MAPK → HIF-1α” oncogenic signaling axis, which may be one of the key upstream mechanisms by which hypoxia drives gastric cancer progression.

As a key transcription factor, HIF-1α activation drives metabolic reprogramming in gastric cancer cells. It does this by upregulating a series of glycolysis-related genes, such as glucose transporter 1 (GLUT1), hexokinase 2 (HK2), pyruvate kinase M2 (PKM2), and lactate dehydrogenase A (LDHA). This causes tumor cells to favor glycolysis even under aerobic conditions - a phenomenon known as the Warburg effect (Zhang and Yuan, 2026). This provides core molecular evidence that hypoxia leads to metabolic reprogramming. This metabolic reprogramming provides energy (ATP) and biosynthetic precursors for rapid tumor growth (Zhu et al., 2026), and the metabolites it produces, such as lactate, create an acidic tumor microenvironment. Glycolysis has become a central feature of cancer cell metabolic reprogramming (Dong et al., 2025). HIF-1α also regulates downstream targets such as CCL7 and KIAA1199, forming a HIF-1α/CCL7/KIAA1199 axis that accelerates glycolysis and promotes tumour progression (Mi et al., 2023). At the transcriptional level, HIF-1α regulates FOXO4 expression. FOXO4 binds to the LDHA promoter and modulates its activity, thereby affecting the glycolytic rate (Wang et al., 2021). Feedback mechanisms, such as the N4-acetylcytidine (ac4C)-mediated NAT10/SEPT9/HIF-1a positive feedback loop, stabilize SEPT9 mRNA under hypoxia and amplify HIF-1α signaling, further exacerbating glycolytic dependency (Yang et al., 2023). Additionally, genes like AAED1 are involved in HIF-1α-mediated glycolytic activation under hypoxia, supporting tumor cell proliferation and survival (Zhang et al., 2018).

Under the influence of these metabolic changes, hypoxia also drives immune cells toward aerobic glycolysis, producing lactate that activates myeloid-derived suppressor cells (MDSCs), upregulates programmed death ligand 1 (PD-L1) (Xiang et al., 2025), promotes T cells (Tregs) and M2 macrophages, and suppresses CD8^+^/NK cells, creating an immunosuppressive microenvironment (Wu et al., 2025).

In addition to HIF-1α, HIF-2α not only regulates cancer cell survival and proliferation but also induces angiogenesis, evades immune surveillance, promotes epithelial-to-mesenchymal transition, and facilitates distant metastasis (Keith et al., 2011; Zhao et al., 2015). Consequently, HIF affects the progression of gastric cancer by regulating the proliferation, metastasis, apoptosis, drug resistance, angiogenesis, stemness, and metabolism of gastric cancer cells. Many small molecule drugs that inhibit the progression of gastric cancer through HIF have been found in basic experiments (Li et al., 2022).

However, hypoxia does not act in isolation. Rather, it interacts with *H. pylori* infection, dietary habits, and the gut microbiota to form a complex network that collectively drives the high incidence of gastric cancer in high-altitude regions. Research has elucidated the molecular mechanisms by which *H. pylori* contribute to gastric carcinogenesis, including the induction of chronic inflammation, genetic alterations, and disruption of cell signaling pathways (Polk et al., 2010; Ferreira et al., 2018). Sustained *H. pylori* stimulation under hypoxic conditions significantly influences the function of GES-1 cells. This stimulation induces epithelial-mesenchymal transition and contributes to the propensity for malignant transformation of gastric cells (He et al., 2024). METTL14 was downregulated in *H. pylori*-infected gastric mucosal epithelial cells, thereby positively regulating HIF-1α expression. Functional experiments showed that overexpressing HIF-1α or knocking down METTL14 enhanced the PI3K/AKT pathway activity. This drives a series of malignant transformations, including EMT, as well as cell proliferation, migration, and invasion (An et al., 2024). Meanwhile, the dietary pattern in high-altitude regions, consisting primarily of pickled and smoked foods, increases nitrite intake (He et al., 2025), thereby exacerbating gastric mucosa damage.

Under the combined influence of environmental and pathogenic factors, systemic changes also occur in the gut microbiota metabolites. Non-targeted metabolomics analysis based on LC-MS revealed elevated levels of glycylproline, glycine, and hydroxyisocaproic acid in the faeces of high-altitude gastric cancer patients; cytidine levels, 5’-methylthymidine, and trehalose decreased. Furthermore, these faecal metabolites were significantly correlated with key serum biomarkers, suggesting the existence of a “microbiota-metabolism-serum” interactive axis jointly regulated by environmental and pathological factors (Zhu et al., 2025). Histopathological findings corroborated this metabolic disturbance: the prevalence of chronic atrophic gastritis, intestinal metaplasia, and moderate-to-severe glandular loss was significantly higher among high-altitude residents, with more severe damage to the gastric body and cardiac region (Recavarren-Arce et al., 2005). This indicates that the cumulative effects of multiple factors, including hypoxia, infection, diet, and microbial metabolic imbalance, have an additive effect on gastric mucosal damage and drive the progression of the disease towards gastric cancer.

In summary, chronic hypoxia reshapes tumor biology and weakens immune surveillance via the HIF signaling pathway. Meanwhile, *H. pylori* infection and an unhealthy diet, coupled with gut microbiota metabolic imbalance, further exacerbate local inflammation and damage. Together, these factors accelerate the Correa cascade (atrophy → intestinal metaplasia → dysplasia → carcinogenesis) and form a vicious cycle that drives the high incidence of gastric cancer and poor prognosis in high-altitude regions.

## 4 Prevention and treatment - recommendations for the prevention and management of high-altitude gastric disorders

High-altitude gastric disorders encompass a wide range of conditions, including functional disorders, organic diseases, and malignant tumors. As these conditions all carry significant risks, it is crucial to develop a comprehensive strategy that includes behavioral interventions, pharmacological prevention and treatment, and disease management.

### 4.1 Prevention

#### 4.1.1 Graded ascent

Individuals with a history of altitude-related illness are more susceptible to AMS. Therefore, the best strategy is to acclimatize gradually to high altitudes. When planning to spend the night at elevations above 2,500-3,000 m, it is recommended that daily elevation gain be limited to <500 m and that one rest day be scheduled for every 3–4 days of ascent (Davis and Hackett, 2017; Derstine et al., 2024; Luks et al., 2024).

#### 4.1.2 Screening and eradication of *H. pylori*

An unhealthy or unbalanced diet can lead to dysbiosis of the gut microbiota. *H. pylori* infection may further exacerbate this condition (Rueda-Robles et al., 2021). In high-altitude regions, individuals who consume raw or cold foods are more prone to gastric issues. Therefore, reducing the intake of such foods after rapid ascent can mitigate gastric damage. Given the high prevalence of *H. pylori* infection in these regions, implementing standardized screening and eradication measures could help to reduce the incidence of gastric diseases.

### 4.2 Pharmacological treatment

The treatment focuses on managing gastric symptoms. Acute high-altitude sickness can affect gastric motility, in which case prokinetic agents may be helpful. Mucosal protectants such as misoprostol, sucralfate, and bismuth subsalicylate can alleviate acute gastric mucosal damage. At the same time, using antioxidants and anti-inflammatory drugs may improve treatment outcomes. In addition, certain traditional Chinese medicines have also demonstrated significant efficacy in treating high-altitude-related gastric disorders. For instance, agarwood (Aquilaria sinensis) has confirmed antibacterial and antioxidant properties (Xie et al., 2024), and studies have shown its effectiveness in alleviating bile reflux gastritis and hypoxic gastric damage (Ma et al., 2025; Wang et al., 2025). Rapid ascent to high-altitude regions alters the gut microbiota composition, thereby reducing the protective effect of SCFAs on the intestine. Therefore, regulating SCFA production by modulating gut microbiota composition can promote overall host health. **Table 1** lists medications used to treat high-altitude gastric disorders and their mechanisms of action (Kangwan et al., 2019; Adinortey et al., 2020; Irons et al., 2020; Teng et al., 2020; Wu et al., 2020; Chiu et al., 2021; El-Salhy et al., 2021; Li et al., 2021; Simon et al., 2021; Liu et al., 2022; Mougey et al., 2022; Qi et al., 2023; Ahmed, 2024; Tae et al., 2024; Ma et al., 2025; Wang et al., 2025).

**Table 1:** Medications for treating high-altitude gastric disorders and their mechanisms of action.

Drug type	Drug name	Efficacy and mechanism	Application scope
Alleviate oxidative stress and inflammatory responses	Agarwood essential oils	Suppresses apoptosis in gastric mucosal cells by modulating the Wnt/β-catenin pathway. Additionally, it reduced hypoxia-induced lipid peroxidation and partially restored dysbiosis and metabolic disturbances caused by hypoxia (Ma et al., 2025; Wang et al., 2025).	Protects mucosal membranes, provides antioxidant benefits, and regulates gut flora. It directly targets hypoxic gastrointestinal injury and bile reflux gastritis.
	Astragalus membranaceus	Inhibits the differentiation of hematopoietic stem cells towards the erythroid lineage and decreases the production of peripheral blood erythrocytes and hemoglobin by reducing the expression of HIF-1α, EPO, VEGFA, and Gata-1 mRNA. It also alleviates inflammatory responses in organs by downregulating the expression of IL-6, TNF-α, and IFN-γ mRNA (Liu et al., 2022).	Reduces inflammation by specifically targeting high-altitude polycythemia (HAPC) and the gastric mucosal inflammation and damage associated with it.
Gastrointestinal motility enhancers	Atractylenolide-1	Activation of the SCF/C-kit signaling pathway modulates the response to oxidative stress, inhibits apoptosis in Cajal interstitial cells, and enhances gastric motility (Li et al., 2021).	Improves gastrointestinal motility and provides antioxidant benefits. Alleviates gastrointestinal motility disorders caused by hypoxia at high altitudes.
	Azithromycin	Stimulates motilin receptors to induce substantial propulsive gastric contractions (Mougey et al., 2022).	Promotes gastric emptying, for treating gastroparesis and symptoms of delayed gastric emptying after rapid ascent to high altitudes.
	Metoclopramide	Inhibits stimulation of the chemoreceptor trigger zone (CTZ) in the medulla by antagonizing dopamine receptors in both central and peripheral systems, thereby alleviating nausea symptoms (Irons et al., 2020).	Relieves nausea and vomiting symptoms associated with acute mountain sickness.
	Mosapride	Stimulates the gastrointestinal 5-HT4 receptors, promoting the release of acetylcholine, thereby enhancing gastrointestinal motility and coordination (Tae et al., 2024).	Promotes peristalsis throughout the gastrointestinal tract, suitable for gastrointestinal motility insufficiency associated with high-altitude environments.
	Cinitapride	Exhibits dual effects as a 5-HT receptor agonist and a dopamine receptor antagonist (Qi et al., 2023).	Promotes peristalsis throughout the entire gastrointestinal tract.
Drugs for Protecting and Repairing the Mucosal Barrier	Misoprostol	Protects the gastric mucosa by inhibiting gastric acid secretion, increasing mucosal blood flow, and promoting mucus/bicarbonate secretion (Ahmed, 2024).	Classic mucosal protectant for the prevention and treatment of stress ulcers.
	Sucralfate/Bismuth Subcitrate Potassium	Enhances Occludin expression to strengthen tight junctions. Regulate the gastrointestinal microbiota (Teng et al., 2020).	Traditional mucosal protectant, indicated for adjunctive treatment of high altitude-related gastric mucosal injury.
	Dissotis rotundifolia	Reduces bleeding, edema, and leukocyte infiltration while elevating glutathione levels, lowering MDA levels, enhancing CAT and SOD enzyme activity, and protecting the gastric mucosa (Adinortey et al., 2020).	Exerts gastric protective effects through antioxidant mechanisms.
	Rosmarinic Acid	Reduces ulceration rates, decrease gastric acid secretion, and lower acidity levels, thereby protecting the gastric mucosa through anti-inflammatory and anti-apoptotic mechanisms (Kangwan et al., 2019).	Anti-inflammatory, anti-secretory, with distinct gastric mucosal protective activity.
Drugs for Regulating the Gut Microbiome	Polyphenols	Promotes the production and growth of beneficial bacterial species to counteract gastrointestinal damage (Chiu et al., 2021).	Regulates gut flora and provides antioxidant benefits. As part of dietary interventions, it helps maintain intestinal homeostasis.
	Probiotics, Prebiotics, and Synbiotics	Prevents dysbiosis and alleviate symptoms of gastrointestinal reactions (Simon et al., 2021).	Maintaining microbial stability by directly addressing intestinal dysbiosis caused by high-altitude hypoxia is a crucial preventive and therapeutic strategy.
	Fecal Microbiota Transplantation	Directly modulates the composition and stability of the gut microbiota, influencing the production of short-chain fatty acids (SCFAs) (El-Salhy et al., 2021).	Restoring gut microbiota. This represents a potential intervention strategy for high-altitude gastrointestinal disorders associated with dysbiosis.
	Rhein	Modulating the gut microbiota indirectly alters intestinal purine metabolism, thereby alleviating intestinal inflammation (Wu et al., 2020).	Acting through the “microbiota-metabolite” axis, it provides a therapeutic approach for high-altitude intestinal disease.

### 4.3 Standardized treatment for gastric diseases

Standardized treatment pathways exist for gastric diseases. Individuals with confirmed gastric conditions should undergo standardized treatment before ascending to high altitudes to minimize further damage to the gastric mucosa. Treatment primarily involves acid suppression and gastric mucosal protection. Beyond these treatments, a recent animal study indicated that low-frequency pulsed electromagnetic fields (PEMFs) can penetrate stomach tissues to alleviate HSU symptoms and promote the regeneration of disrupted tissues. This suggests the clinical potential of PEMF therapy for treating HSU (Jiao et al., 2019).

## 5 Conclusions

High-altitude gastric disorders are multifactorial pathophysiological processes involving hypoxia and regulatory dysfunctions across multiple systems, including the mucosal barrier, HIF-mediated metabolic signaling pathways, gastrointestinal hormones, motility, the gut microbiota, and neuroendocrine function. In severe cases, these disorders may lead to complications such as gastrointestinal bleeding and perforation. Therefore, risk assessment and proactive intervention are crucial for individuals traveling to high-altitude regions. Future research should focus on identifying intervention targets and mechanisms that can modulate the gut microbiota to prevent gastric damage, as well as novel differential biomarkers for high-altitude gastric injury. Additionally, research should explore the specific active ingredients in traditional Chinese medicine for treating high-altitude gastric disorders, which could provide new insights for developing more precise and effective prevention and treatment strategies.
